# Conserved Expression of Nav1.7 and Nav1.8 Contribute to the Spontaneous and Thermally Evoked Excitability in IL-6 and NGF-Sensitized Adult Dorsal Root Ganglion Neurons In Vitro

**DOI:** 10.3390/bioengineering7020044

**Published:** 2020-05-16

**Authors:** Rahul R. Atmaramani, Bryan J. Black, June Bryan de la Peña, Zachary T. Campbell, Joseph J. Pancrazio

**Affiliations:** 1 Department of Bioengineering, University of Texas at Dallas, Richardson, TX 75080, USA; rxa162330@utdallas.edu (R.R.A.); bjb140530@utdallas.edu (B.J.B.); 2 Center for Advanced Pain Studies, University of Texas at Dallas, Richardson, TX 75080, USA; JuneBryan.delaPena@utdallas.edu (J.B.d.l.P.); Zachary.Campbell@utdallas.edu (Z.T.C.); 3 Department of Biological Sciences, University of Texas at Dallas, Richardson, TX 75080, USA

**Keywords:** inflammation, dorsal root ganglion, nociceptor, IL-6, NGF, Nav1.7, Nav1.8, microelectrode arrays

## Abstract

Sensory neurons respond to noxious stimuli by relaying information from the periphery to the central nervous system via action potentials driven by voltage-gated sodium channels, specifically Nav1.7 and Nav1.8. These channels play a key role in the manifestation of inflammatory pain. The ability to screen compounds that modulate voltage-gated sodium channels using cell-based assays assumes that key channels present in vivo is maintained in vitro. Prior electrophysiological work in vitro utilized acutely dissociated tissues, however, maintaining this preparation for long periods is difficult. A potential alternative involves multi-electrode arrays which permit long-term measurements of neural spike activity and are well suited for assessing persistent sensitization consistent with chronic pain. Here, we demonstrate that the addition of two inflammatory mediators associated with chronic inflammatory pain, nerve growth factor (NGF) and interleukin-6 (IL-6), to adult DRG neurons increases their firing rates on multi-electrode arrays in vitro. Nav1.7 and Nav1.8 proteins are readily detected in cultured neurons and contribute to evoked activity. The blockade of both Nav1.7 and Nav1.8, has a profound impact on thermally evoked firing after treatment with IL-6 and NGF. This work underscores the utility of multi-electrode arrays for pharmacological studies of sensory neurons and may facilitate the discovery and mechanistic analyses of anti-nociceptive compounds.

## 1. Introduction

Nociceptor cell bodies reside in the dorsal root ganglion (DRG). Their fibers innervate the dermis, internal organs, and the viscera. In addition to a critical role in detection of noxious external stimuli in their peripheral receptive field, nociceptors transmit information from the periphery to the dorsal horn of the spinal cord in the form of all-or-nothing action potentials [1]. The generation and propagation of such action potentials are driven by voltage-gated sodium channels (VGSCs) [2]. Three VGSC α-subunits are preferentially expressed in C-nociceptive subpopulations of small-medium diameter DRG neurons, namely, Nav1.7, Nav1.8, and Nav1.9 [3]. As a result, the dysregulation of Nav1.7 and Nav1.8 subtypes in specific pain modalities has been linked to the maintenance of inflammatory pain states [4].

Inflammatory mediators released at the site of injury result in peripheral activation and the sensitization of nociceptors due to their effects on a multitude of ion channels, including sodium channels [5]. Under chronic pain conditions, maladaptive changes in sodium ion channel subtypes in nociceptive populations have been extensively documented in pre-clinical models of inflammatory pain in vivo. Injury generally results in increased accumulation of Nav1.7 and Nav1.8 protein [5,6,7,8,9,10]. In the acute phase, mitogen-activated protein kinases (MAPKs), stimulated by pro-nociceptive cytokines, have been shown to directly modulate nociceptive excitability by either altering the gating properties, in the case of Nav1.7 [11], or current density, in the case of Nav1.8 [12]. These findings suggest that the pharmacological disruption of Nav1.7 and Nav1.8 post-translational modification, activity, or expression may ameliorate specific pain phenotypes. Consistent with this notion, patients with loss of function mutations in the voltage-gated sodium channel Nav1.7 are insensitive to pain [13]. Similarly, knockdown of Nav1.8 in pre-clinical models can reverse a specific model of neuropathic pain [14]. Specifically, the role of interleukin-6 (IL-6) and nerve growth factor (NGF) in inflammatory pain has been extensively established and shown to alter Nav1.7 and Nav1.8 activity under pathological conditions [15,16,17,18]. NGF, for example, has been shown to directly upregulate Nav1.7 protein content and increase the level of mRNA transcripts [19]. Additionally, the downstream activation of signal transduction molecules by IL-6 has been reported to regulate the expression and phosphorylation of Nav1.7, altering its excitability [17]. Similarly, Nav1.8 expression has been observed to increase in vivo [20,21] and in vitro [22] following application of NGF. Ceramide, a signal transducer of NGF, increases Nav1.8 current density [23]. In total, given the importance of IL-6 and NGF in inflammatory pain mechanisms and the functional consequences of altering the expression and function of Nav1.7 and Nav1.8, further investigation of these inflammatory mediators in altering DRG neuron excitability may be warranted.

Acutely dissociated adult DRG neurons have demonstrated the conserved expression of Nav1.7 and Nav1.8 in DRG cultures in vitro, showing sodium channel modulation and hyperexcitability induced by inflammatory mediators [2,24,25]. These approaches have largely used dissociated small- and medium-gauged naïve rodent DRG neurons for single cell patch-clamp recordings. However, most of these measurements are limited to acutely dissociated tissue (14–20 hours after harvest) and performed at room temperature with a short exposure time to the specific chemokine under investigation [3,9,26,27,28,29]. As a result, low-throughput patch-clamp measurements are not well-positioned for long-term (e.g., days in duration) studies that may be critical to understand the underlying mechanisms involved in inflammatory cytokine sensitization which occur over chronic time periods [5,6,10,30,31,32,33,34,35,36]. Substrate integrated microelectrode arrays (MEAs), alternatively, provide non-invasive and long-term measurements recorded simultaneously from a large population of cells. The use of an MEA platform may allow the development of in vitro phenotypic screening assays that utilize cellular excitability for screening potent small molecule inhibitors against “pain” channels and support efforts in identifying analgesic compounds [37,38,39,40,41,42,43]

Embryonic DRG neurons have been successfully cultured on substrate integrated microelectrode arrays (MEAs) and develop spontaneously active networks [44]. However, reliance on embryonic tissue is problematic due to the differential expression with adult tissue and/or responsiveness of ion channel subtypes to pharmacological treatments [44,45]. Long-term in vitro studies spanning days require a culture approach where key receptors are stably expressed. In some culture models, receptor expression degrades [46] or becomes dysregulated [47], raising the question of whether or not adult DRG express the VGSCs over time in culture, consistent with that which we expect from adult tissue in vivo.

Recently, we have demonstrated that adult DRG neurons cultured on multi-well MEAs are spontaneously active and are responsive to treatment with inflammatory cytokines such as interleukin-6 (IL-6), eliciting increased spontaneous activity and stimulus-evoked responses [15,48]. In the present study, we investigate the role of Nav1.7 and Nav1.8 after chronic exposure to IL-6 and NGF in vitro using adult DRG cultured on MEAs. We demonstrate that adult DRG neurons cultured for extended periods in vitro retain expression of relevant VGSCs to pain and bioelectrical activity, shows modulation to specific antagonists, an effect distinct from that observed using cultured cortical neurons. Both subtypes are required for sensitization by IL-6 and NGF but display differential effects on thermally evoked firing events. The study highlights the utility of adult DRG cultures on MEAs as a potential phenotypic screening tool for antagonists to sodium channels that are relevant to pain.

## 2. Materials and Methods 

### 2.1. Primary Adult DRG Culture

All surgical procedures were carried out in accordance with the University of Texas at Dallas’s Institutional Animal Care and Use Committee. Institute for Cancer Research (CD-1) male mice (4–6 weeks old; Envigo) were utilized for DRG tissue extraction, as described in detail previously [49]. Briefly, mice were deeply anesthetized with 3% isoflurane and euthanized via cervical dislocation. Spinal columns were extracted, hemisected, and DRGs were isolated from central/peripheral roots and stored in ice-cold Hanks Balanced salt solution (HBSS). Tissue was dissociated as previously described with minor modifications [15]. An enzyme buffer consisting of 2 mg/ml collagenase and 0.1 mg/mL DNase was added to pooled tissue and allowed to incubate at 37 °C for 40 min. After 40 min, 100 μL of 1× papain solution was added and the tissue was gently triturated using a fire polished pasture pipette. The cells were isolated via centrifugation (300 *g* for 10 min) and resuspended in a fresh medium consisting of DMEM/F-12 + glutamax + 10% fetal bovine serum (FBS) + 1% penicillin/streptomycin (PS) + 5 ng/mL GDNF. A total of 10,000 viable neurons were plated on multi-well MEAs (Axion BioSystems, Atlanta, GA, USA) pre-treated with 50 μg/mL poly-D-lysine (overnight) followed by surface coating of 20 μg/mL laminin (2 hours). Cultures were maintained at 37 °C, 10% CO_2_, and 95% humidity and medium was exchanged every 48 hours. After non-neuronal populations reached a confluence of approximately 90%, medium was supplemented with mitotic inhibitors uridine (17.5 μg/mL) and 5-fluoro-2’-deoxyuridine (7.5 μg/mL) for the remainder of the culture. 

### 2.2. Primary Embryonic Cortical Culture 

Murine-derived cortical networks were derived from embryonic age (E15-E18) mice. Time pregnant female mice (ICR-CD1, Envigo RMS Inc, Indianapolis, IN, USA) were deeply anesthetized followed by euthanasia via cervical dislocation. Individual embryos were obtained via cesarean section, removed from amniotic sacs, and stored in ice-cold HBSS. Cortical neurons were dissected and dissociated from 3–6 embryos, as described in detail previously [50]. Briefly, frontal cortices were surgically sectioned and pooled in an enzyme buffer consisting of 0.1 mg/mL DNAase and 100 µL of 1× papain reconstituted in HBSS and incubated at 37 °C for 30 min. Tissue sections were homogenized via mechanical trituration using a fire-polished pasture pipette and cells were isolated via centrifugation (300 *g* for 10 min). A total of 90,000 viable cells were plated on a pre-treated multi-well MEAs as described previously and maintained in Dulbecco’s Modified Eagle’s Medium supplemented with 5% horse serum, 5% FBS, and 1% PS. Medium was exchanged after 48 hours, after which serum was removed to prevent over proliferation of non-neuronal cells and maintained by 50% medium exchanges for at least 21 days in vitro. 

### 2.3. Extracellular Recordings

Spontaneous and evoked extracellular recordings were performed with 48-well plate MEAs (Axion Biosystems, Atlanta, GA, USA) using the Axion Maestro MEA recording system (Axion Biosystems, Atlanta, GA, USA), as described previously [15]. Briefly, extracellular voltage recordings were carried out at 12.5 kHz sampling rate from a total of 768 available substrate integrated microelectrodes. Continuous data were filtered using a 1-pole Butterworth band pass filter (200–3000 Hz) and individual spikes were detected using a 5.5 σ adaptive threshold method. For analysis, only electrodes were considered for analysis when a mean firing rate of at least 1 spike/min was detected during the recording session. As such, the analysis considers both spontaneous and evoked increase in activity from previously spontaneous “active” channels and previously quiescent channels which became active in response to treatment with inflammatory cytokines (see below) and/or a temperature stimulus. Additional analysis was carried out in NeuroExplorer (Nex Technologies, Madison, AL, USA) and AxIS Metric. 

### 2.4. Pharmacology

Before exposure, all pharmacological compounds were reconstituted in either complete medium, water, or DMSO at a stock concentration of ≥100×. Before the addition of compounds, a baseline recording of 30 min was acquired. For exposure to IL-6 and NGF or vehicle (water), bolus volumes were added simultaneously to treatment groups (100 ng/mL IL-6 + 100 ng/mL NGF) and spontaneous recordings were acquired at the following discreet time points: 3 hours, 48 hours, and 72 hours. At each timepoint, a temperature challenge was introduced using the Environmental Control system in AxIS 2.3. Briefly, at the end of the associated 30 min baseline recording, the temperature was increased at 0.5 °C /min until 42 °C and held at 42 °C for 5 min, while recording changes in activity. To determine the pharmacological responsiveness to Nav1.7 (Huwentoxin-IV 30 nM), Nav1.8 (A-803467 300 nM), and Nav1.1/1.3 (ICA-121431 23 nM) blockers, respective compounds were added to treatment groups that were previously incubated with IL6 and NGF or vehicle for 72 hours followed by a 10-min recording session. For analysis, total spike count from n = 6 wells per treatment was compared to a 30-min baseline recording taken immediately prior to addition. The log spike count for each treatment well was compared to the respective baseline and data are represented as the average percentage change from baseline. In all experimental cases, deionized (DI) water was used as the vehicle for IL-6 and NGF treatment and DMSO was used as the vehicle for the voltage gated sodium channel antagonists. 

### 2.5. Immunostaining and Image Analysis

Post treatment with either IL-6 or vehicle, day in vitro (DIV) 13–15 primary DRG cultures were fixed with 4% paraformaldehyde for 10 min at room temperature (RT) followed by a triple wash with ice-cold PBS. Cells were permeabilized by 0.3% Triton X-100 for 30 min at RT. Following permeabilization, non-specific binding sites were blocked with 2% normal goat serum (NGS) for 30 min, and samples were incubated with the following primary antibodies: Anti-NeuN (1:500, ABN91, Millipore Sigma), Anti-Nav1.7 (1:200, ab65167, Abcam), and Anti-Nav1.8 (1:500, ab93616, Abcam) for 18 hours. The following day, species specific secondary, goat anti-chicken Alexa Fluor 647 (1:200, ab150171, Abcam), goat anti-mouse Alexa Fluor 546 (1:200, A-11030, Thermofisher) and goat anti-rabbit Alexa Fluor 488 (1:200, ab150077, Abcam) antibodies were incubated for 1 hour at RT. Confocal images were acquired at 20× magnification using an inverted Nikon Eclipse Ti (Nikon, Tokyo, Japan) microscope. All confocal images of stained samples were acquired with a 20× objective with a numerical aperture (NA) of 0.5 and a calculated depth of focus of 3.6 µm. To determine the percentage of Nav1.7- and Nav1.8-expressing neurons in IL-6- or vehicle-treated groups, a minimum of three regions of interest (ROIs) were selected from a total of n = 6 wells (n = 3 IL-6, n = 3 vehicle). Multi-channel images were processed in ImageJ (NIH). Briefly, a user-defined threshold was applied to all channels and neuronal somata were manually counted based on maximum intensity projections. For the normalized mean fluorescence intensity of associated Nav1.7 and Nav1.8 expression post treatment with IL-6 and NGF (or vehicle), circular ROIs were drawn across a minimum of n = 60 somata, and the signal intensity of Nav1.7 and Nav1.8 was measured using ImageJ (NIH). For all experiments, only presumptive nociceptors were selected (diameter ~ 20 µm), and cells were identified as positive for Nav1.7 and/or Nav1.8 if the mean fluorescence intensity was ≥2.5× then the average intensity of the background. 

### 2.6. RNA Isolation and Quantitative Real-Time PCR (qRT-PCR)

Total RNA from DIV13 DRG cultures treated with IL-6 (n = 6 wells) or vehicle (n = 6 wells) was isolated and purified using the GeneJET RNA Purification Kit (Cat#: K0731, Thermo Scientific). RNA concentration was determined using Thermo Scientific NanoDrop One Microvolume UV-Vis Spectrophotometer. A total of 1 μg of total RNA was reverse transcribed to cDNA using ImProm-II Reverse Transcription System (Cat#: A3800, Promega), according to the manufacturer’s instructions. The cDNA was amplified by a set of sequence-specific primers (Custom Oligos Service, Sigma Aldrich) and detected with SYBR Green reagent (Cat#: 1725120, BioRad) using a CFX Connect Real-Time PCR Detection System (Cat#: 1855200, BioRad). The qRT-PCR reactions were performed in triplicate. The cycling conditions included a denaturing step at 94 °C for 1 min, followed by annealing at a primer-specific temperature for 1 min, and elongation at 72 °C for 45 s. Previously validated primer sequences against Nav1.7 and Nav1.8 in mouse DRG neurons have been used [51], and are as follows: 

Nav1.7 (Scn9a) forward TCCTTTATTCATAATCCCAGCCTCAC

Nav1.7 (Scn9a) reverse GATCGGTTCCGTCTCTCTTTGC

Nav1.8 (Scn10a) forward ACCGACAATCAGAGCGAGGAG

Nav1.8 (Scn10a) reverse ACAGACTAGAAATGGACAGAATCACC

GAPDH forward CTAGGACTGGATAAGCAGGGC

GAPDH reverse GCCAAATCCGTTCACACCGA. 

Fold changes in gene expressions were quantified using the ΔΔCt method, where Ct is defined as the threshold cycle (Livak and Schmittgen, 2001). Values were normalized to GAPDH.

### 2.7. Statistical Analysis 

All statistical tests and visualization of datasets were carried out in OriginPro 2019 (OriginLab, Northampton, MA, USA). To compare differences between two sample proportions, a test of proportions z-test was used. A two-sample *t*-test was performed to compare means of two groups to test the hypothesis of no difference between treatment and vehicle groups. For all conditions, a *p* value < 0.05 was considered as statistically significant. Unless otherwise stated, all descriptive statistics are represented as mean ± standard error of the mean (SEM). 

## 3. Results

### 3.1. IL-6 and NGF Increase Spontaneous and Stimulus Evoked Excitability

**Figure 1:**
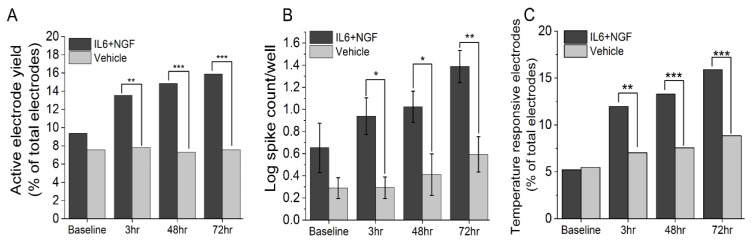
IL-6 and NGF induce an acute and persistent increase in excitability and thermally mediated evoked responses in adult DRG neurons in vitro. (**A**) Percentage of active electrodes post treatment with 100 ng/mL IL6 + 100 ng/mL NGF or vehicle over 3–72 hours period. (**B**) Log spike count per well post treatment over a 3–72 hours period. (**C**) Percentage of temperature responsive electrodes post treatment over a 3–72 hours period. Responsiveness to temperature was defined as an electrode exhibiting a mean firing rate (MFR) of ≥ 2× that of baseline. Temperature stimulus was introduced in the form of base plate heating to a temperature of 42 °C and held for a period of 5 min. An active electrode was defined as an electrode exhibiting a mean firing rate (MFR) of at least 1 spike/min. Error bars are represented as mean ± standard error of the mean (SEM). * *p* < 0.05, ** *p* < 0.01, and *** *p* < 0.001.

To assess changes in the excitability of adult DRG neurons in vitro in response to known inflammatory mediators, DIV13-DIV15 cultures were treated with a combination of 100 ng/mL IL-6 and 100 ng/mL NGF or vehicle and spontaneous and evoked activity were assessed at 3 hours, 48 hours, and 72 hours timepoints. Treatment with the combination of IL-6 and NGF led to increased instances of spontaneously active neurons in terms of active electrodes (IL-6+NGF: 3-, 48-, 72-hour: 13.5%, 14.8%, 15.9%; Vehicle: 3-, 48-, 72 hour: 7.8%, 7.3%, 7.6%; test of proportions z-test, *p* < 0.05, Figure 1A) and a greater number of spikes compared to vehicle-treated groups (IL-6+NGF: 3-, 48-, 72 hour: 0.94 ± 0.17, 1.02 ± 0.14, 1.39 ± 0.15; Vehicle: 3-, 48-, 72-hour: 0.29 ± 0.10, 0.41 ± 0.19, 0.59 ± 0.16; two-sample *t*-test, *p* < 0.05; Figure 1B). To determine whether nociceptor-evoked response to noxious temperature is potentiated by IL-6 and NGF, we measured the percentage of temperature-responsive electrodes (mean firing rate ≥ 2× of baseline at 42 °C) and compared treatment to vehicle groups. We observed a significant increase in thermally evoked responses in IL-6 and NGF treated groups at all time points assayed (3–72 hour) (IL6 + NGF: 3-, 48-, 72-hour: 11.9%, 13.3%, 15.9%; Vehicle: 3-, 48-, 72 hour: 7.0%, 7.6%, 8.8%, test of proportions z-test, *p* < 0.05; Figure 1C.). These data suggest that IL-6 and NGF evoke a significant increase in the recruitment of previously quiescent neurons and and an overall increase in the spike count. Additionally, they may mediate thermal hypersensitivity at acute and persistent time periods in vitro.

### 3.2. IL-6 and NGF-Mediated Hyperexcitability Is Attenuated by Nav1.7 and Nav1.8 Antagonists

**Figure 2:**
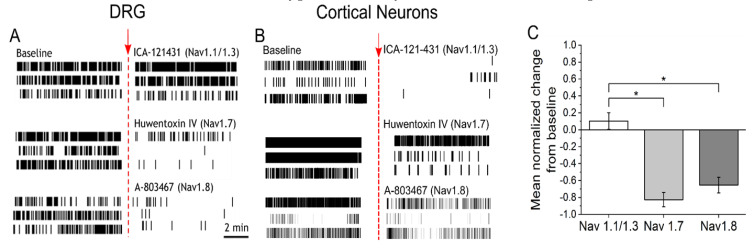
Potent and selective antagonists of Nav1.7 and Nav1.8 inhibit spontaneous activity in sensitized DRG cultures (**A**) Representative raster plots of three active electrodes in baseline (left) and after addition (right) of either 23 nM ICA-121431 (top), 30 nM huwentoxin-IV (HWTX-IV) (middle), or 300 nM A-803467 (bottom). Inhibitors of sodium channel subtypes were administered on cultures sensitized with IL6 + NGF for a period of 72 hours. (**B**) Representative raster plots of three active electrodes in baseline and equivalent treatments in DIV21 embryonic cortical cultures. (**C**) Mean normalized change in firing rate from baseline of DRG cultures after addition of ICA-121431 (Nav1.1/1.3), HWTX-IV (Nav1.7), or A-803467 (Nav1.8). Error bars are represented as mean ± standard error of the mean (SEM). * *p* < 0.05, ** *p* < 0.01, and *** *p* < 0.001.

**Figure 3:**
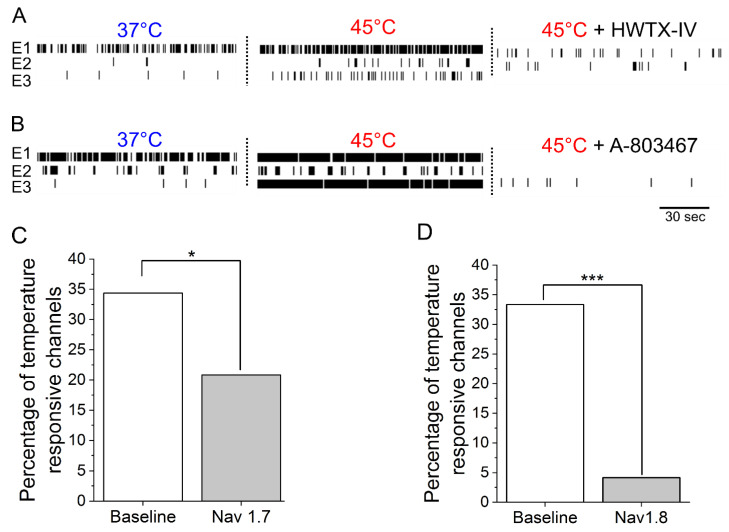
Potent and selective antagonists of Nav1.7 and Nav1.8 inhibit thermally evoked responses in sensitized adult DRG cultures. (**A**) Raster plots of three representative electrodes at 37 °C (left), 45 °C (middle), and after the addition of 30 nM HWTX-IV. (**B**) Raster plots of three representative electrodes at 37 °C, 45 °C, and after the addition of 300 nM A-803467. Summarized percentage of temperature responsive electrodes at baseline and after the addition of (**C**) Nav1.7 blocker (30 nM HWTX-IV) and (**D**) Nav1.8 blocker (300 nM A-803467).

To determine if sensitization by NGF and IL-6 requires Nav1.7 and Nav1.8, adult DRG neurons incubated with IL-6 and NGF for 72 hours were treated with 30 nM huwentoxin IV (HWTX-IV) (Nav1.7 blocker) or 300 nM A-803467 (Nav1.8 blocker). Additionally, 23 nM ICA-121431, a potent inhibitor of central nervous system (CNS)-specific Nav1.1/1.3 channel subtypes, was administered as a negative control. At nanomolar concentrations, both HWTX-IV and A-803467 were potent at inhibiting spontaneous DRG activity (HWTX-IX: 83% ± 9% inhibition, n = 6 wells; A-803467: 65% ± 9%, n = 6 wells, *p* < 0.05 compared to negative control, ICA-121431; Figure 2A,C). In contrast, the inhibition of CNS-specific Nav1.1/1.3 channel subtypes by ICA-121431 had little to no effect on spontaneous DRG activity (Figure 2A,C). To further elucidate the subtype-selective modulation of nociceptive-specific ion channels in our DRG tissue preparation, DIV21 embryonic cortical networks were administered with HWTX-1V, A-803467, and ICA-121431. As expected, ICA-121431 caused strong inhibition of spontaneous cortical activity (92 ± 3% inhibition, n ≥ 3 wells), whereas HWTX-IV (16% ± 5% inhibition, n ≥ 3 wells, *p* = 0.3) and A-803467 (5% ± 0.4% inhibition, n ≥ 3 wells, *p* = 0.1) failed to significantly reduce activity (Figure 2B). Additionally, both HWTX-IV and A-803467 reduced 35% of previously temperature-responsive channels to 20% and 4%, respectively, in DRG cultures—both statistically significant reductions (*p* = 0.03 and *p* < 0.0001, respectively, test of proportions z-test; Figure 3A–D). Taken together, these data indicate that the pharmacological profiles of Nav1.7 and Nav1.8 are maintained in adult DRG neurons in vitro. We find that both channels contribute to the IL-6- and NGF-mediated increase in hyperexcitability and stimulus evoked responses.

### 3.3. Nav1.7/1.8 Expression is Unchanged after 72 Hour IL-6 and NGF Treatment

**Figure 4:**
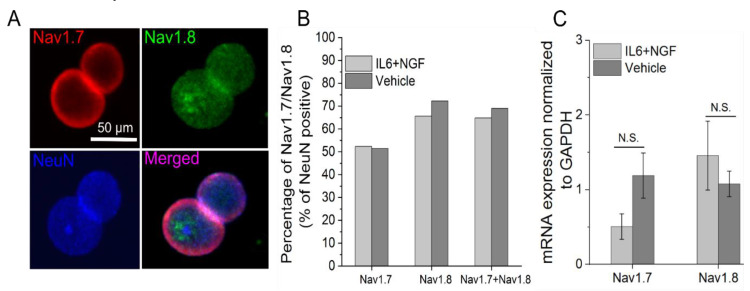
Nav1.7/1.8 expression is maintained in adult DRG neurons in vitro and is unchanged by treatment with IL-6 and NGF. (**A**) Representative immunofluorescence images of adult DRG neurons stained for Nav1.7 (top left; red), Nav1.8 (top right; green), and neuronal marker NeuN (bottom left; blue). (**B**) Percentage of Nav1.7, Nav1.8, and Nav1.7/1.8 co-expressing neurons following exposure to IL-6 and NGF for 72 hours or vehicle. (**C**) mRNA expression of Nav1.7 and Nav1.8 normalized to glyceraldehyde 3-phosphate dehydrogenase (GAPDH) following exposure to IL6 and NGF for 72 hours or vehicle. Errors are expressed as mean ± STD.

Next, we asked whether the observed increase in excitability induced by IL-6 and NGF treatment is due to the increased accumulation of Nav1.7/1.8 protein. DIV 13–15 adult DRG neurons were incubated with 100 ng/mL IL-6 and 100 ng/mL NGF for 72 hours, and Nav1.7/1.8 expression was measured via immunostaining (Figure 4A.). Small DRG neurons (≤30 μm) with a signal intensity of Nav1.7/1.8 above a define threshold (≥2.5× mean background intensity) was 52% and 65% after IL-6 and NGF treatment (Figure 4B). The percentage of Nav1.7/1.8 positive neurons in treatment groups did not increase compared to vehicle (Vehicle, Nav1.7: 51%, Nav1.8: 72%; *p* = 0.79, *p* = 0.03, respectively; Figure 4B.). Furthermore, quantification of the mean fluorescence intensity revealed a similar expression of the sodium channel subtypes in treatment and vehicle groups (IL6 + NGF, Nav1.7: 3.16 ± 0.41 A.U., Nav1.8: 3.50 ± 0.18 A.U., Vehicle, Nav1.7: 3.75 ± 1.21, Nav1.8: 4.20 ± 1.71, n = 3 wells, *p* = 0.46, *p* = 0.52, respectively; two sample *t*-test). The observed absence of modulation of ion channel abundance post treatment with IL-6 and NGF was further supported by mRNA expression studies. Using quantitative real-time PCR (qRT-PCR) (Figure 4C), we found that Nav1.7/1.8 transcripts were not significantly different between treatment and vehicle groups (IL6 + NGF, Nav1.7: 0.51 ± 0.38, Nav1.8: 1.46 ± 0.18, Vehicle, Nav1.7: 1.19 ± 0.1, Nav1.8: 1.08 ± 0.02, n = 6 wells, *p* = 0.2, *p* = 0.09, respectively; two sample *t*-test; data not shown). Therefore, the data suggest that both mRNA and protein expression of Nav1.7/1.8 were unchanged after acute (72 hours) treatment with IL-6 and NGF. We conclude that expression levels are unlikely to explain increased activity of Nav1.7/1.8 after sensitization by NGF and IL-6.

## 4. Discussion

The present study demonstrates that the expression and pharmacological profiles of VGSC subtypes, Nav1.7 and Nav1.8, are maintained in adult DRG neurons in vitro and may partially contribute to the acute and persistent changes in excitability and thermally evoked responses post treatment with IL-6 and NGF. Additionally, we have demonstrated that such maladaptive hyperexcitability is readily abrogated by pharmacological blockade of Nav1.7 and Nav1.8. The observed increase in excitability is unlikely related to regulation of Nav1.7 and Nav1.8 expression as the mRNA and protein levels are unaltered after sensitization. 

Nav1.7 channels produce fact activation and inactivation, slow repriming currents and yield large ramp currents in response to small, slow depolarizations. Thus, they are poised to amplify generator potentials in nociceptors in response to a stimuli, such as the activation of ligand-gated ion channels, and increases the probability of depolarizing the membrane, giving rise to neuronal firing in response to ion channel fluctuations or natural stimuli. In contrast, the fast repriming, depolarized activation, and slow inactivating kinetics of Nav1.8 are implicated in the repetitive firing in DRG neurons [2,52]. Therefore, in the present study, the conserved expression profiles and unique biophysical properties of preferentially expressed Nav1.7 and Nav1.8 channels may be critical determinants in the generation of the ectopic spontaneous activity in cultured DRG neurons in vitro.

The role of Nav1.7 and Nav1.8 in inflammatory pain and thermal hyperalgesia has been previously demonstrated in vivo using pre-clinical models. For example, ablation of the gene that encodes Nav1.7, *Scn9a*, [53] or conditional knock out Nav1.7-expressing Nav1.8 neurons reduced inflammation-mediated pain behavior induced by formalin and Complete Freund’s Adjuvant (CFA). Furthermore, the presence of thermal hyperalgesia was diminished in Nav1.7 null-mice Similarly, Nav1.8 null mice display attenuation of NGF-induced thermal hyperalgesia [54]. Consistent with in vivo findings, we report that pharmacological antagonists of Nav1.7/1.8 led to a decrease in thermally mediated evoked responses as measured during a noxious temperature range (42 °C) induced in vitro. Although the effect of radiant heat used in determining thermal nociceptive thresholds in animal models is procedurally and mechanistically distinct wherein, the onset of the noxious temperature is on the order of seconds [36,55], the underlying mechanism of encoding functional temperature sensitivity by nociceptors to noxious temperature was conserved and successfully recapitulated in vitro. More importantly, treatment with antagonists of Nav1.7/1.8 reduced the observed thermal sensitivity in a manner consistent with its role in thermal hyperalgesia in vivo [36]. This result is perhaps surprising given the pleiotropic nature of the mediators given their effects on cap-dependent and Poly(A)-dependent translation [56,57,58,59,60,61,62,63,64,65,66,67,68]. In contrast to several prior reports, we find that NGF and IL-6 have no impact on the expression of Nav1.7/1.8. CFA and NGF result in an increase in the expression of Nav1.7 and Nav1.8 in vivo [6,7,59,60,61,62]. However, the time of administration (24 h – 1 week) and concentration (5 µg NGF) differ from the present in vitro exposure (100 ng/mL NGF). Moreover, the inflammatory state induced by CFA may include a myriad of inflammatory cascades and mediators including serotonin, histamine, and adenosine, which may not be recapitulated in vitro. While chronic exposure to inflammatory mediators has been shown to modulate protein accumulation, we favor a model wherein the acute effects of nociceptive sensitivity are due to direct activation of the ion channel isoforms. For example, it has been shown that 100 ng/mL acute incubation (30 min) of NGF with DRG neurons increased an initial response to capsaicin and brief pre-treatment with anisomycin causes a rapid increase in Nav1.8 current density in a p38-dependent manner [12,63]. This direct activation, presumably due to the phosphorylation of TRPV1 via MAPK, is fully consistent with the present findings, where the acute modulation of spontaneous activity was readily observed after 3-hour treatment with IL6 and NGF. Additionally, it has been shown that the addition of IL-6 to cultured nociceptors results in a direct association of ERK and Nav1.7, modulating hyperexcitability by the direct phosphorylation of Nav1.7 [11,15,17].

The present findings highlight the utility of adult DRG neurons as a tissue source for screening potential therapeutics that modulate Nav1.7 and Nav1.8. Prior studies have utilized embryonic DRG tissue which may not be amenable to studying NGF-induced sensitization, as high doses of the neurotrophic factor are strictly required to maintain viability in culture [44]. Moreover, the expression profiles of Nav1.7 and 1.8 are significantly different among adult and embryonic tissue [64]. Notably, the culture of dissociated sensory neurons and the maintenance of gene expression profiles in vitro is non-trivial. For example, Roland et al. (1997) reported that dissociated cultures of embryonic DRG neurons expressed trkA and trkC receptors dissimilar to those found in intact DRG explants, suggesting the differential expression of genes that are repressed under physiological conditions which may manifest in vitro [65]. A similar dysregulation of receptor expression in vitro has been observed in aortic endothelial cells [47] and neuronal cells [46]. In contrast, the mRNA expression profiles of Nav1.7 and Nav1.8 in the present study were maintained for at least 13 days in vitro, and are consistent with profiles in acutely dissociated adult mouse DRG neurons shown previously [32]. Additionally, our histological study demonstrated a high percentage of Nav1.8-expressing neurons in combination with Nav1.7 (60–70%) which is consistent with previous observations in DRG tissue [2,3,41], wherein 88% of cultured DRG tissue was described as nociceptive. An in-depth characterization of Nav1.7 and Nav1.8 expression in neurochemically distinct nociceptive populations demonstrated an enriched expression of Nav1.7 and Nav1.8 in NF200−/TrkA+ and NF200−/TrkA- adult DRG populations compared to non-nociceptive NF200+/TrkA− subtypes, suggesting preferential expression of these isoforms in peptidergic and non-peptidergic populations [66]. Similarly, Black et al. (2012) observed Nav1.7 expression in 65% IB4+ and 58% of CGRP+ neurons, with only 15% colocalizing with NF200+ positive neurons. Taken together, the present data are consistent with previous findings that Nav1.7 and Nav1.8 isoforms are preferentially expressed in nociceptive populations and such histiotypic profiles can be expected to be maintained in long-term, in vitro cultures of adult DRG neurons. This suggests that our present culture methods provide a reliable platform for the study of Nav1.7 and Nav1.8 for long-term measurements and highlight the utility of phenotypic screens using adult DRG neurons subjected to inflammatory mediators that promote chronic changes in physiological activity.

It is worth mentioning that the cause of ectopic spontaneous discharge of afferent C-fiber nociceptors in vivo is mechanistically distinct from the in vitro spontaneous firing of uninjured DRG neurons from control or naïve animals, with the former being related to a specific inflammatory or injury-related mechanism leading to sensitization [67,68]. Microneurographic recordings of single C-fibers in neuropathic pain conditions have revealed increased excitability which may account for ongoing pain and confers a pathological, maladaptive condition [69]. The spontaneous activity of uninjured DRG neurons in vitro is caused by ion channel states being stochastic, leading to fluctuations in current through the ion channel, and creating a source of intrinsic excitability which triggers spontaneous action potentials [70]. Notably, in the context of developing a DRG-neuron cell-based assay, the generation and maintenance of such baseline spontaneous activity is imperative to acquiring a clinically relevant phenotypic endpoint for screening compounds which specifically alter excitability [71].

Although the use of adult murine-derived DRG neurons is useful for the study of molecular, cellular, and bioelectrical mechanisms involved in the generation and maintenance of inflammatory pain states, disadvantages have been described previously in the translatability of rodent models to human clinical pain [72]. Notably, the expressions of Nav1.7 and Nav1.8 channel isoforms are higher in human nociceptors compared to murine-derived nociceptors [73]. However, such discrepancy in expression profiles does not necessarily reduce the importance of a DRG-based assay to model hyperexcitability and screen potential antagonists in vitro, since the effect of several pain-related molecules are conserved across species [74].
